# Editorial: Molecular and cytogenetic research advances in human reproduction

**DOI:** 10.3389/fendo.2022.1107903

**Published:** 2022-12-09

**Authors:** Ihtisham Bukhari, Rick Francis Thorne, Qinghua Shi

**Affiliations:** ^1^ Henan Key Laboratory of Helicobacter pylori, Microbiota and Gastrointestinal Cancers, Marshall Medical Research Center, Fifth Affiliated Hospital of Zhengzhou University, Zhengzhou, China; ^2^ Translational Research Institute, Henan Provincial and Zhengzhou City Key Laboratory of Non-coding RNA and Cancer Metabolism, Henan International Join Laboratory of Non-coding RNA and Metabolism in Cancer, Henan Provincial People’s Hospital, Academy of Medical Sciences, Zhengzhou University, Zhengzhou, Henan, China; ^3^ School of Environmental and Life Sciences, University of Newcastle, Callaghan, NSW, Australia; ^4^ Division of Reproduction and Genetics, First Affiliated Hospital of USTC, Hefei National Laboratory for Physical Sciences at Microscale, School of Basic Medical Sciences, Division of Life Sciences and Medicine, Biomedical Sciences and Health Laboratory of Anhui Province, University of Science and Technology of China, Hefei, China

**Keywords:** molecular reproduction, human reproduction, male infertility, female infertility, reproductive diseases

Infertility is a frequently diagnosed medical condition affecting over 15% of couples worldwide (1–3). It has diverse causes, including genetic and environmental factors (4, 5) with dozens of molecular and cytogenetic factors being identified associated with male and/or female infertility. Mainly, male infertility is caused by chromosomal abnormalities including an abnormal number or structure of sex chromosomes or autosomes (6, 7). Other than such gross chromosomal changes, single-gene mutations have recently received attention as a cause of infertility (8–11). Indeed, hundreds of mutations have been identified in men suffering from reproductive defects (12, 13), and more or less a similar number of mutations have been identified in infertile women (14, 15). Such mutations occur in genes responsible for germ cell development and other reproductive processes (16–19).

Animal models mimicking human mutations have been generated to study the mechanisms underlying the effects of the gene mutation (20). Different studies reveal their contribution at various stages of the spermatogenesis or oogenesis (21). Nevertheless, thousands of functionally relevant genes are expressed in human testes and ovaries, and malfunctioning even a single gene can potentially cause infertility (22). Moreover, routine testing for male infertility is limited to chromosome aberrations and Y-chromosome microdeletions (23, 24). However, more and more attention is paid to DNA mutations including the roles of gene expression regulators such as 3’UTR sequences (25–27). Since the impact of mutations in gene 3’UTR sequences may not be adequately recognized in current sequencing analyses, a potentially massive gap between fertility genetics and its clinical applications has arisen.

The current Research Topic aimed to collect clinical and basic research studies reporting advancement in cytogenetic, molecular, and clinical aspects of human reproduction. After rigorously reviewing submitted articles, the current volume presents an authoritative collection of nine articles exploring new dimensions of human reproduction.

Firstly, Gao et al. introduces the role of KCNQ1 in sperm development and maturation. KCNQ1 is predominantly expressed and localized to the head and tail of sperm; its silencing causes male infertility by significantly reducing sperm motility and acrosome reaction. Alongside sperm motility, sperm morphology has long been known to be critical for successful fertilization. The study by Khan et al. presented an analysis of multiple morphological abnormalities of sperm flagella (MMAF) in two Pakistani families with several infertile brothers. Intriguingly, they found that the spermatozoan fibrous sheaths were disorganized and the central singlet of microtubules was missing. Extensive analyses revealed that the cause of MMAF involved DNAH1 mutations. Galdon et al. successfully isolated the cells from the testicular tissues of individuals with Klinefelter Syndrome (KS) and maintained the culture over 110 days. Cells with abnormal and normal karyotypes were found in testicular cells after culture. These findings could be helpful in the diagnosis and development of fertility therapies for KS patients, such as *in vitro* spermatogenesis or transplantation of SSC *in vivo*.

Testes are essential for male reproduction; different genes and signaling pathways control testes’ cellular physiology and morphology. Wang et al. reviewed the role of androgen receptor signaling in spermatogenesis. As known, the androgen receptor signaling regulates biological processes in Sertoli cells and germ cells. Functionally, the androgen receptor helps in the proliferation and maturation of Sertoli cells, self-renewal, and differentiation of spermatogonia stem cells. Additionally, it maintains the integrity of the blood-testis barrier to smoothen the overall process of spermatogenesis. Similarly, Yu et al. compiled the functions of estrogen receptors in female endometrium. Mainly, estrogen induces the proliferation of endometrium mucosa through interacting with estrogen receptors, an essential step in normal menstruation and pregnancy. The abnormal expression of estrogens or their receptors could cause endometriosis (EMT), endometrial hyperplasia (EH), endometrial cancer (EC), and infertility. Therefore, it is suggested that future studies focus on evaluating new therapeutic strategies that target specific ERs and their related growth factor signaling pathways. Liu et al. investigated increases in ApoE in follicular fluid (FF) and the possible relationship between ApoE and EMT in 217 Chinese women (111 healthy controls and 106 EMT patients). Higher expression of ApoE was detected in the FF of EMT patients; however, ApoE affected the quality of blastocysts while did not change levels of fertility hormones. Thus, ApoE levels could only be used as a predictor of EMT, but further details studies are advised. On the other hand, Yu et al. determined that the HOXA10 substantially overexpressed in the ovarian endometriotic cysts stromal cell (OESC) and further uncovered that HOXA10 and its interacting genes are critical for cholesterol biosynthesis in endometrial stromal cells. To find the genetic difference in natural and IVF-ET conceived pregnancies, Yang et al. used placental tissues from both pregnancies for genetic profiling. They identified 12 differentially expressed miRNAs and 258 genes in IVF-ET placental tissues significantly enriched in angiogenesis, pregnancy, PI3K-Akt, and Ras signaling pathways. These findings provide a resource of potential molecular markers to assess the association between placental function and safety in IVF-ET offspring. Finally, Tan et al. explored the relationship between recurrent miscarriages and ovarian reserves. For this purpose, they recruited women with a history of one miscarriage, two and three, or more miscarriages. They found that with the increased number of recurrent miscarriages, the levels of anti-Müllerian hormone, antral follicle counts, and ovarian reserves significantly decreased.

## Conclusion

Recent advances in next-generation sequencing (NGS) technologies have brought a paradigm shift in investigating rare and common human disorders. The ability cost-effectively to generate genome-wide sequencing data with in-depth coverage in a short time frame is replacing approaches that focus on specific regions for gene discovery and clinical testing. Articles included in the current research topic enlighten us about various aspects of human reproduction, such as the roles of different genes, hormones, and technologies. Isolation of normal testicular cells from Klinefelter syndrome could bring the hope of conceiving a child. However, it is too early to predict, but it is an essential step toward developing infertility cures.

## Author contributions

All authors listed have made a substantial, direct, and intellectual contribution to the work and approved it for publication.
